# Clinical ethics committees in nursing homes: what good can they do? Analysis of a single case consultation

**DOI:** 10.1177/09697330211003269

**Published:** 2021-07-13

**Authors:** Morten Magelssen, Heidi Karlsen

**Affiliations:** Centre for Medical Ethics, University of Oslo, Norway

**Keywords:** Case study methods, clinical ethics, clinical ethics committees, clinical ethics support, empirical approaches, nursing homes

## Abstract

**Background:**

Ought nursing homes to establish clinical ethics committees (CECs)? An answer to this question must begin with an understanding of how a clinical ethics committee might be beneficial in a nursing home context – to patients, next of kin, professionals, managers, and the institution. With the present article, we aim to contribute to such an understanding.

**Aim:**

We ask, in which ways can clinical ethics committees be helpful to stakeholders in a nursing home context? We describe in depth a clinical ethics committee case consultation deemed successful by stakeholders, then reflect on how it was helpful.

**Research design:**

Case study using the clinical ethics committee’s written case report and self-evaluation form, and two research interviews, as data.

**Participants and research context:**

The nursing home’s ward manager and the patient’s son participated in research interviews.

**Ethical considerations:**

Data were collected as part of an implementation study. Clinical ethics committee members and interviewed stakeholders consented to study participation, and also gave specific approval for the publication of the present article.

**Findings/results:**

Six different roles played by the clinical ethics committee in the case consultation are described: analyst, advisor, support, moderator, builder of consensus and trust, and disseminator.

**Discussion:**

The case study indicates that clinical ethics committees might sometimes be of help to stakeholders in moral challenges in nursing homes.

**Conclusions:**

Demanding moral challenges arise in the nursing home setting. More research is needed to examine whether clinical ethics committees might be suitable as ethics support structures in nursing homes and community care.

## The case

*An elderly man had been a permanent resident of a municipal nursing home since the death of his wife a few years ago. He had a diagnosis of dementia. He was somewhat physically fit, however, and walked with the aid of a rollator walker. Before retirement, he had a physically demanding occupation, and he had always been active and fond of outdoor life. He took long, daily walks from the nursing home, often heading towards the city centre and crossing several roads with traffic. Next of kin had mounted a GPS on his walker; the device sent location information every 20 minutes which nursing home staff had access to. The institution’s policy had been to allow the patient to take his walks alone but to fetch him back after approximately two hours. If staff attempted to accompany the patient, the patient expressed dissatisfaction; he was also often unwilling to accompany staff back to the nursing home when they retrieved him.*

## Introduction

*Clinical ethics support services (CESS)* aid in the handling of ethical challenges in healthcare.^1–3^ The *clinical ethics committee (CEC)* is a kind of CESS which has been established in many hospitals in many Western countries.^4^ Nursing homes (residential long-term care), however, are seldom served by CECs. The United States is an exception; here, CECs have a long history in nursing homes.^5–7^ Neither in the United States, however, are there any recent systematic overviews of the prevalence and activities of nursing home CECs. A recent review of published research on CECs in residential long-term care found only 13 primary studies (nine from the United States, four from European countries).^5^ There were no rigorous evaluation studies,^i^ but some studies that describe the activities that the CECs perform. These find that the trio of activities typical for hospital CECs – case consultations, policy review and ethics education for staff – are common activities also for nursing home CECs. The authors of the review called for more research on roles and impact of CECs on patients and institutions.^5^

CECs are multi-professional committees charged with improving ethics competence and analysing and giving advice on ethical challenges from daily practice. When deliberating on ethics cases, they typically involve professionals, next of kin or the patient in the deliberations, and produce a written case report detailing the case, the values involved, and potential lines of action. The CEC may offer advice, but the advice is typically not binding for the professionals.

Ought nursing homes to establish CECs? In order to answer this question, a thorough-going understanding of what benefits a CEC might bring in a nursing home context – to patients, next of kin, professionals, managers, and the institution – is a necessary first step. With the present article, we aim to contribute to such an understanding. We ask: In which ways can CECs be helpful to stakeholders in a nursing home context? To shed light on this, we describe in some depth a single CEC case consultation which stakeholders deemed successful, and we emphasize the impact of the consultation on the stakeholders. We then reflect on the roles played by the CEC in this case and the potential benefits of case consultations in nursing home CECs.

The case springs from an evaluation study in which our group studies the establishment of four CECs in community (municipal) care, covering nursing homes as well as other community (municipal) health and care services. Our group provides implementation support and studies aspects of the establishment and activities of the CECs for 2.5 years. The study is presented in a separate paper.^8^

## Methods

### Data sources

The article is a case study, presenting in anonymized form a case discussed in 2019 in a municipal CEC serving the nursing home where the case originated. We draw on four data sources: The CEC’s written case report; the CEC’s brief self-evaluation form which the CEC completed as part of the evaluation study; a research interview with the nursing home’s ward manager; and a research interview with the patient’s son who was the closest next of kin. The two research interviews were performed by the second author. They were semi-structured and aided by interview guides created for interview series with professionals and next of kin respectively. Interviews were audiotaped, then transcribed verbatim.

### Analysis

The facts of the case, presented at the outset of the article, were summarized from the description in the CEC’s case report. The description of the CEC consultation summarizes the case report with supplemental information from the CEC’s self-evaluation form. The research interviews were read by both authors independently, with a view to identifying different aspects of impact of the case consultation on the stakeholders. Quotes from the interviews are shown in order to validate key interpretations. The first author then sketched potential roles played by the CEC in the case. These were then refined through both authors’ hermeneutic reflection process and discussions, and after a discussion of a draft version presented to colleagues at our department. In addition to the findings from the interviews, the reflection was inspired by a previous analysis of hospital CECs’ roles in priority setting issues which identified seven roles played by the CECs.^9^ Although we aimed for the roles to be constructed inductively from the material, the authors’ pre-understanding of what takes place in CEC case deliberations necessarily also played a role in the process; we attempted to make this pre-understanding explicit and to challenge it in light of the findings. In the final account of the roles played by the CEC we briefly connect each role to an example from the findings, providing warrant for each role. As we draw on a single case only, the account of the roles we were able to make was necessarily somewhat brief.

### Research ethics

Data were collected as part of a study which has been evaluated by the Data Protection Official at the Norwegian Centre for Research Data (project no. 56714). According to the Norwegian system, no further ethics approval was required. CEC members and interviewed stakeholders were informed of the study in writing and gave written, informed consent. These informants then gave specific, written approval for the publication of the present article, and they have been offered to read through the article before publication. The patient was deemed not to have capacity to consent; his son has given consent as the closest next of kin. Stakeholders have been anonymized in the article as far as possible.

## Results

### The CEC’s case consultation

The recently established CEC, comprising 12 members, served all health and care services in a Norwegian municipality. The CEC was contacted by the nursing home’s ward manager, who wanted a broad discussion and an outside view on the case (presented at the outset of the article). Prior to the contact the manager had discussed the issue with the patient’s son several times. The manager had judged it as most appropriate to allow the patient to take walks, yet worried about the risk of harm and potential consequences. Four CEC members met with the manager, three healthcare professionals involved in daily clinical work with the patient, and the patient’s son. The patient was not present. Before the meeting, the CEC head had spoken to the manager and the son and informed about the CEC. The case deliberation took place at the nursing home, lasted 75 minutes and was structured by the so-called CME (Centre for Medical Ethics) model for ethics deliberation (Table 1). The CME model has six steps and builds on ideas from discourse ethics. It is designed to bring forth morally relevant features of a case and structure the discussion.^10^ The model’s different steps are referred to in parentheses throughout the presentation of the case consultation.

**Table 1.** The CME model for ethics deliberation.

1. What is the moral problem?
2. What are the facts of the case?
3. Who are the stakeholders, and what are their views and interests?
4. Which moral norms, principles, values and virtues, and laws and guidelines are involved?
5. What are the possible lines of action?
6. Analysis and preferred line of action

CME: Centre for Medical Ethics.

The CEC defined the moral problem as «ought staff to limit the patient in taking walks alone?» (step 1 (Table 1)). The patient was described as easily bored inside the nursing home and dissatisfied with current restrictions on his walks (step 3). The son claimed that the way he knew his father before the onset of dementia, the father would have accepted the risks to himself involved in the unsupervised walks. Among staff, several expressed concerns that the patient might be injured, through falling into the water, being hit by a car or getting lost and becoming confused and scared. They also worried about their personal responsibility should anything happen. There were different viewpoints among staff about the appropriate policy. The patient’s son was worried that the father might come to harm, yet wanted him to live well and in accordance with his own preferences. The son was unsure of what the best policy would be, but emphasized that the walks were important for his father’s well-being. The son realized that the situation was difficult for staff and for the manager in particular.

When making values and norms explicit (step 4), the CEC and participating stakeholders made reference to the four traditional principles of medical ethics.^11^ Applying these principles to the case, it was argued that *nonmaleficence* implied protecting the patient from injury, yet also avoiding dissatisfaction from inactivity if restrictions on his walks were continued or increased. *Beneficence* was involved because being outdoors, taking walks, and reaching the destinations he wanted to were experienced by the patient as delightful. After completed trips, he was markedly calm and satisfied. *Respect for autonomy* was clearly also pertinent. The patient was determined, wanted to decide for himself and expressed dissatisfaction with being too closely monitored by staff. Less obviously, *justice* was also relevant. Staff spent quite some time looking for and retrieving the patient. Less time and attention would then be available for other patients. Furthermore, the patient was brought back by taxi, involving significant expenses for the institution. A further ethical principle, *quality of life*, was also invoked. The freedom to take walks was argued to be important for the patient’s physical and mental well-being. Walks gave content to his days, and he had been used to being active and in good shape all his life.

From the perspective of law (step 4), the CEC’s lawyer highlighted that the municipality more than individual staff would carry legal responsibility should injury befall the patient. Furthermore, the law could not settle the case but would primarily require that any policy settled upon would have been well-meditated and documented.

Discussing potential policies (steps 5 and 6), the CEC concluded that quality of life and patient autonomy were the concerns carrying the most significant weight in the situation. This judgement justified continuing a policy of letting the patient take unsupervised walks; thus accepting some risk, in the interest of giving the patient a broader sense of freedom and self-determination. This should, however, be balanced with staff’s regular reassessment of the patient’s condition and capabilities. In the case of deterioration, the policy should be reviewed.

### What did the case consultation lead to?

For the patient, the consultation led to increased freedom in line with his expressed preferences. A policy was established that he was free to take walks, and for 3 h at a time as opposed to 2 h previously.

For the manager and other professionals, the consultation amounted to a confirmation that they had identified morally relevant considerations and weighed them in a way that could be defended. The judgement that the patient’s freedom ought to prevail had now been confirmed by an independent, external, and multidisciplinary body established by the municipality, and given an explicit ethical justification. This upshot was particularly significant for the ward manager, who experienced having the final word on this decision and similar decisions as a significant responsibility:

It’s a little easier now to say that, relax now, let him walk, in a way. [Because] we have thought about this…The day it happens, it happens. We have evaluated, we have considered the risks we can consider. We have discussed it in every direction we can, we have thought ethically, professionally sound, and everything. So, it does become a bit, it does become a support for me as a leader, to in a way hear it from all sides.

The manager also appreciated the written report, which she described as good and important. The report had been read and discussed by all staff involved in the patient’s care, and then also put in the patient records. This resolved disagreement among staff; all were subsequently comfortable with the policy:

[The staff not present at the meeting] were also very pleased afterwards, both to hear that we had analyzed what we should, and not the least that next of kin had been there and said what they had said.

Finally, the manager also appreciated how the consultation had made values and ethics explicit; she stated: ‘it is an important part which we might forget a bit when we are there in the direct care’. The systematic approach afforded by the CME deliberation model was also appreciated.

For next of kin, the meeting allowed the son to explain how his father had lived his life before the onset of illness, and this was appreciated. The son described how his father had always preferred an active lifestyle, and claimed that this explained his present preferences also:

So, he is the kind of fellow who was very, was very much on the move…I explained a lot around that…so he has been very active, or he was perhaps both restless and active. And one sees that now, that now it’s a somewhat different thing, but still all that remains.…So, I think this affected them [i.e., staff present at the CEC consultation] in the way that, oh yeah, that explains a bit.…It explains a bit why he, in a way, just leaves.…It was really very okay to get to explain it, because they were of course not aware of it.

In the meeting, the son also received some information about his father’s care which he had not known. The son also commended the systematic approach to making the ethics of the case explicit. In the interview, the son stated that it was positive that the staff had shown uncertainty and doubt:

No, I actually think that’s positive [i.e., that staff had acknowledged uncertainty]. Because it shows you that one has a commitment and an interest in ensuring that residents are well taken care of, I think.

The seriousness with which they took the situation and the very fact that a CEC consultation was held to discuss it strengthened his impression that his father was well taken care of. Besides, the son stated that the policy agreed upon and the thorough process made him and the other next of kin feel secure.

You feel in a way that you’ve had it evaluated, professionally and ethically, by people who know this much better than I…which makes it more safe, I feel it’s more safe for us also.

The new policy also relieved them of some guilty conscience for not visiting the father more often, as they now were assured that the father’s needs would be met. Finally, in case of any injury, it would be significant that the case had been so thoroughly discussed in the CEC and joint agreement reached:

If something were to happen in five months, then I think it’s very good for everyone involved to, in a way, have had it up for discussion. And one has, in a way, reached an agreement.

The CEC stated in the self-evaluation report that they considered the consultation successful in several respects, including the involvement of stakeholders. The presence of next of kin and his explanation of the patient’s values was emphasized as particularly helpful for the deliberation, as was the presence of a municipal lawyer.

By reflecting on the experiences reported by the different stakeholders, we have formulated six roles played by the CEC in the case deliberation. The roles are listed and explained in Table 2. The CEC acted as an *analyst* in formulating an explicit moral problem and showing, with the aid of different steps of the ethics reflection model, how moral principles were relevant. As an *advisor*, the CEC justified and recommended a course of action. The *support* of the CEC as a forum for external validation of the decision reached by the clinicians and manager, was important. Acting as a *moderator*, the CEC consultation brought important stakeholders together in a moral dialogue. The CEC was also a *builder of consensus and trust*, in that it helped overcome disagreement and insecurity among staff and helped stakeholders settle on an agreed policy and trust each other. Finally, the CEC also acted as a *disseminator* of relevant knowledge of ethics and law, exemplified by the clarification of legal responsibilities in case of injury, and by the sharing of the case report also with staff not present at the meeting.

**Table 2.** Six roles played by the CEC in the case.

Role	Explanation
Analyst	Making the moral problem and involved values and principles explicit, and showing how values and principles justify courses of action
Advisor	Justifying and recommending the best course of action
Support	Providing a forum for external validation and support of the formal decision-maker’s decision and sharing the responsibility
Moderator	Bringing stakeholders together in an external forum with ample time, facilitating moral dialogue, leading to new information being made explicit and shared; contribute to a better decision-making process
Builder of consensus and trust	Helping stakeholders overcome disagreement and settle on an agreed policy which all can consider being justified, thus also increasing mutual trust
Disseminator	Sharing pertinent knowledge of ethics, law and regulations with stakeholders

CEC: clinical ethics committees.

## Discussion

### CEC roles and benefits of the consultation

The case concerns a conflict between patient autonomy on the one hand and nonmaleficence and safety on the other, in a patient with reduced decision-making competence. As such the case is a variation of a prevalent, recurrent, significant and often difficult value conflict in health and care services.^12–16^

The experiences and evaluations reported by the stakeholders and summed up in the six roles played by the CEC indicate that the consultation was useful in several respects – and that the CEC can indeed fulfil several vital roles in the nursing home setting. At the same time, the roles are general and not likely to be specific to the nursing home context. The CEC is a forum unlike other meeting places for professionals in their workday, and a forum in which it is natural to include next of kin (and sometimes patients themselves).^4,17^ In the CEC consultation a moral dialogue takes place and values are made explicit. The consultation did not bring assurance against risk and harm, but rather a joint, justified understanding that the value of freedom here outweighed the value of risk reduction. Through this explicit balancing of competing values, the policy has become easier to live with; moral distress is likely to have been mitigated. Notably, the role of ‘support’ involves sharing in and relieving formal decision-makers of some of the burdens of responsibility for decisions potentially of great import. However, the CEC provides advice only, and the formal decision-making is left to the professionals, who remain free to disregard the CEC’s advice. CEC consultations arguably *require* a certain level of trust, yet can also *contribute to* building mutual trust among stakeholders, who get to meet face to face and listen to one anothers’ perspectives.

### The significance of CECs in nursing homes and municipal health and care services

The present case story was chosen because it shows both that nursing home CECs *can* be of significant benefit, and *in what ways* they can be of benefit. In this way, the case indicates that CECs *can* indeed be beneficial for nursing homes, their patients, staff, managers, and next of kin, in the handling of moral challenges.

A further question is whether nursing homes *ought* to establish CECs. This is, of course, a much larger and more complicated question. The present case only contributes to our understanding of how such CECs might operate. Before any general recommendation for the establishment of CECs could be made, one would need to conduct thorough implementation studies, assess not only successful CEC cases but also *typical* cases, and identify costs, burdens and negative consequences. Our group is in the process of conducting such a study, where CECs are established for whole municipalities and not for individual nursing homes.^8^ We would welcome further studies from other countries and settings.

The present case study indicates that CECs can be useful in several different respects, and for many different stakeholders. Case consultation in CECs is a complex activity. This arguably strengthens our claim made previously that a ‘complex intervention’ approach to studying CECs is appropriate.^8^

Although much academic and professional attention has been on high-profile dilemmas in hospitals, community health and care services (including nursing homes) give rise to a host of moral challenges often of great ethical significance and complexity – probably as much so as in hospitals.^18,19^ However, the community setting is marked by less competence and education among staff. In a Norwegian context, middle managers in community care must often shoulder extensive responsibilities, being expected to reach final verdicts to dilemmas – as was illustrated in the case. They must then navigate in the ‘moral space’ for manoeuver created by the barriers erected by health law. Here, ethics support like a CEC would, we propose, sometimes be of significant value to them.^20^

As argued, the case shows CECs potentially to be of value in the nursing home setting. Related community services such as home care, sheltered housing/assisted living facilities, mental health and substance abuse care, school health services, maternal-child and adolescent health stations, and primary care physicians, however, arguably have comparable needs for ethics support.^21–23^ This contention is supported by the nature of the CEC roles identified: The roles are quite general, and thus appear likely to be beneficial across a range of healthcare settings. The question then arises what the proper scope or institutional affiliation is for a CEC in community care. Nursing homes come in many sizes; a small nursing home might not provide enough moral challenges or competence for a dedicated CEC to be worthwhile or feasible. In our implementation project, the CECs are established at the municipal level in four municipalities, and are intended to serve the entirety of the municipality’s health and care services.^8^ The four municipalities have populations of approximately 10,000 to > 80,000.

### CEC versus other ethics support

The CEC is but one of many different models of CESS. A model which is more common in Norwegian community care is the *ethics reflection group*, which is similar to the *moral case deliberation (MCD)* model which originated in The Netherlands.^24^ The ethics reflection group is a lower-level, ‘bottom-up’ forum,^2^ typically consisting of staff working together in the same department/ward. Here, an ethics facilitator leads the discussion of an ethics case drawn from the participants’ practice. In contrast to the CEC, there are typically no external members, and most reflection groups do not involve the next of kin or patients. The group is not expected to document discussions in a detailed case report.

The present case study indicates that a CEC might sometimes provide further benefits compared with ethics reflection groups/moral case deliberation. In our case, the presence of external members and the CEC being an official body in the municipality was significant for conferring some authority on the deliberation and advice, and in giving support to decision-makers. As we saw, the written case report and the presence of next of kin and a lawyer were also significant. The CEC thus performed roles that ethics reflection groups are not as well equipped to fill, because the latter forum typically lacks the institutional authority and experience in including next of kin and patients, and sometimes also the broad competence which includes law and ethics. Arguably, therefore, ethics reflection groups can perform the roles of analysts and advisors, but to a lesser degree the other roles highlighted in our analysis.

### Strengths and limitations

The study highlights the ‘everyday ethics’ in a non-hospital healthcare institution. In-depth analysis of a single case brings out features that might be missed with other methodologies, such as details of how CEC consultations might bring about benefits for stakeholders. Such analyses are therefore helpful supplements to other approaches, especially in the evaluation of a complex intervention. There is a risk of bias in that we as authors here evaluate and analyse a case from a project we ourselves are involved in implementing. Analyses of other and multiple cases would be likely to bring out further and different roles that CECs can play.

## Conclusion

Through analysis of a case deemed successful by stakeholders we have shown ways in which a CEC can be of help in a nursing home setting. In our analysis, the CEC played six different, significant roles – as analyst, advisor, support, moderator, builder of consensus and trust, and disseminator. The case study indicates that CECs might sometimes be of help to stakeholders in the handling of difficult moral challenges in nursing homes. More research is needed to examine whether CECs might be suitable as ethics support structures in nursing homes, community care in general, or municipalities.

## Note

i Janssens et al.^24^ evaluate experiences with moral case deliberation (MCD) and is included in the review; for reasons explained in the Discussion section we distinguish between CECs and MCD.
